# Evolution of Competitive Ability: An Adaptation Speed vs. Accuracy Tradeoff Rooted in Gene Network Size

**DOI:** 10.1371/journal.pone.0014799

**Published:** 2011-04-25

**Authors:** Jacob W. Malcom

**Affiliations:** Integrative Biology, University of Texas at Austin, Austin, Texas, United States of America; University of Oxford, United Kingdom

## Abstract

Ecologists have increasingly come to understand that evolutionary change on short time-scales can alter ecological dynamics (and vice-versa), and this idea is being incorporated into community ecology research programs. Previous research has suggested that the size and topology of the gene network underlying a quantitative trait should constrain or facilitate adaptation and thereby alter population dynamics. Here, I consider a scenario in which two species with different genetic architectures compete and evolve in fluctuating environments. An important trade-off emerges between adaptive accuracy and adaptive speed, driven by the size of the gene network underlying the ecologically-critical trait and the rate of environmental change. Smaller, scale-free networks confer a competitive advantage in rapidly-changing environments, but larger networks permit increased adaptive accuracy when environmental change is sufficiently slow to allow a species time to adapt. As the differences in network characteristics increase, the time-to-resolution of competition decreases. These results augment and refine previous conclusions about the ecological implications of the genetic architecture of quantitative traits, emphasizing a role of adaptive accuracy. Along with previous work, in particular that considering the role of gene network connectivity, these results provide a set of expectations for what we may observe as the field of ecological genomics develops.

## Introduction

Biologists are broadly interested in the drivers of diversity, ranging in scale from nucleotide sequences to the entire biome. One goal is to span across levels of organization: we would like to understand how genes interact with one-another and with environmental inputs to produce phenotypes (the genotype-phenotype map, GPM), and how phenotypes ‘fit’ to the environment (the phenotype-environment map, PEM). Ultimately, we would like to understand the links across all three levels of organization, the genotype-environment map (GEM). Such a goal requires incorporating dynamics from each of the sub-mappings into an over-arching set of expectations. We might ask, for example, how does variation in genetic architecture affect trait evolution, how does trait evolution affect competitive dynamics, and how might competition feed back to alter genetic architecture?

The example of competition is raised because it has a long history in investigations of the maintenance of diversity at the level of the PEM, as exemplified in Hutchinson's “Homage to Santa Rosalia” [1]. Classical ecological analyses, from Lotka-Volterra to Tilman's R* to contemporary models [2]–[5], typically (implicitly) assume that competing species are fixed for the attributes that regulate competitive dynamics, i.e., that ecological dynamics are much faster than evolutionary change. However, as Antonovics noted four decades ago [6], we should expect most ecological changes to be associated with evolutionary change.

Researchers have recently begun to explore and formalize the joint effects of ecological and evolutionary dynamics on species' populations and their communities [7]–[12]. Hairston and colleagues [7] developed several analytical models that incorporate both phenotypic change (evolution) and population change (ecology). They demonstrated that evolutionary change can play a major role in altering population dynamics (as in the case of *Geospiza fortis* populations and evolving bill size), or evolutionary change may play a smaller role (as in the case of *Onychodiaptomus sanguineus* and egg diapause). Fukami and colleagues [13] demonstrated that evolution in *Pseudomonas* communities systematically alters the community structure: a single colonist strain will evolve to occupy several niches, excluding future colonizing strains and changing community structure when compared to a community into which several strains are introduced simultaneously. All of this is to say that the traits that mediate competitive interactions should evolve sufficiently quickly to alter community dynamics.

The rate at which a trait can evolve—which may describe how population dynamics might be affected at different rates of change—is described by the quantitative genetic parameter heritability [14]. One of the advances of the Modern Synthesis was the realization that we did not need to know details of the genetic basis of a trait in order to be able to predict the rate at which the trait will change [15]. All that is needed are estimates of the additive genetic and phenotypic variances of the trait. The heritability of a trait underlying competitive ability should then describe the rate of change of competitive ability. Gomulkiewicz and Holt [16] linked trait heritability to the probability and the rate at which populations recover from sudden environmental change, showing that higher heritability increases the chance of recovery and the rate at which recovery occurs. The predicted U-shaped population decline and recovery pattern expected from their theory has recently been recovered empirically [17]. Now consider extending their result to two initially competitively equivalent species that begin competing for a resource that evolves over time (e.g., a food resource such as phytoplankton that evolves defenses to zooplankton grazing [18]–[20]). We expect that the competitor with the higher heritability for the trait (e.g., tolerance of phytoplankton defenses) to be able to adapt faster and ultimately out-compete the species with lower heritability [21].

Although knowledge of the genetic basis of heritability is not required to make predictions about trait evolution, with the advent of modern genomic and bioinformatic techniques we are beginning to be able to determine the genetic details underlying quantitative traits [22]–[24]. By extension, if a link from genetic sequence to trait heritability exists, there should be a link from genetic sequence to communities by way of traits and their role in mediating competition (i.e., a model that incorporates the GEM). In a previous paper [25], I examined the plausibility of a link between genetic architecture and heritability of a quantitative trait. The results kept with analytical models of biological epistasis and the effects on variance components [26]–[28], such that network structures hide and reveal additive genetic variation so that, even without any environmental variance inputs, heritability is altered. Specifically, I found that smaller networks should tend to have higher heritability than larger networks because hidden additive variance is released and selected on more quickly. In addition, because the quantitative trait is divided among fewer genes, the average effect of a mutation is larger in small gene networks than in large networks. As a result of these two factors, populations with smaller gene networks adapt and recover faster from sudden environmental changes than do populations in which the ecologically-critical trait is underlain by larger networks. By extension, small-network populations persist longer than large-network populations when the environment fluctuates rapidly through time [29]. These results are consistent with previous network-centric research that focused on network connectivity rather than size [30], [31]. Together, they suggest that the competitor with the smaller gene network underlying an ecologically-critical trait should out-evolve and out-compete a species with a larger gene network for the same trait.

Here, I test the hypothesis of maximal fitness arising from minimized network size under the scenario of interspecific competition in a single patch. Two competing species are limited by a resource with two characteristics. First, the resource occurs at a given quantity that limits the total number of individuals in a patch, and the two species are effectively neutral with respect to capitalizing on quantity (i.e., their requirement and impact vectors are identical [32]). Second, the resource has a quantitative value for quality, such as palatability, to which the competing species must adapt in order to maximize their fitness. The quantitative trait, whose value is determined by the gene network, maps to this resource quality. Specifying competition in this way stabilizes the population dynamics relative to a system in which the primary resource is depleted. The ‘focal species’ in the competition possesses a fixed genetic architecture for an ecologically-critical trait (*n* = 16 genes, scale-free network topology, recombination rate  = 0.5, mutation rate = 0.001) while the ‘competitor's’ genetic architecture for the trait varies from 16 to 256 genes, random or scale-free topology, and different recombination and mutation rates. The results highlight a speed-versus-accuracy tradeoff for different networks. Smaller networks confer the advantage of higher adaptive speed in fast-changing environments, whereas larger networks confer greater adaptive accuracy when the environment changes sufficiently slowly. These results provide a set of hypotheses to be empirically tested as we attempt to refine the genotype-phenotype-environment map.

## Results

**Figure 1:**
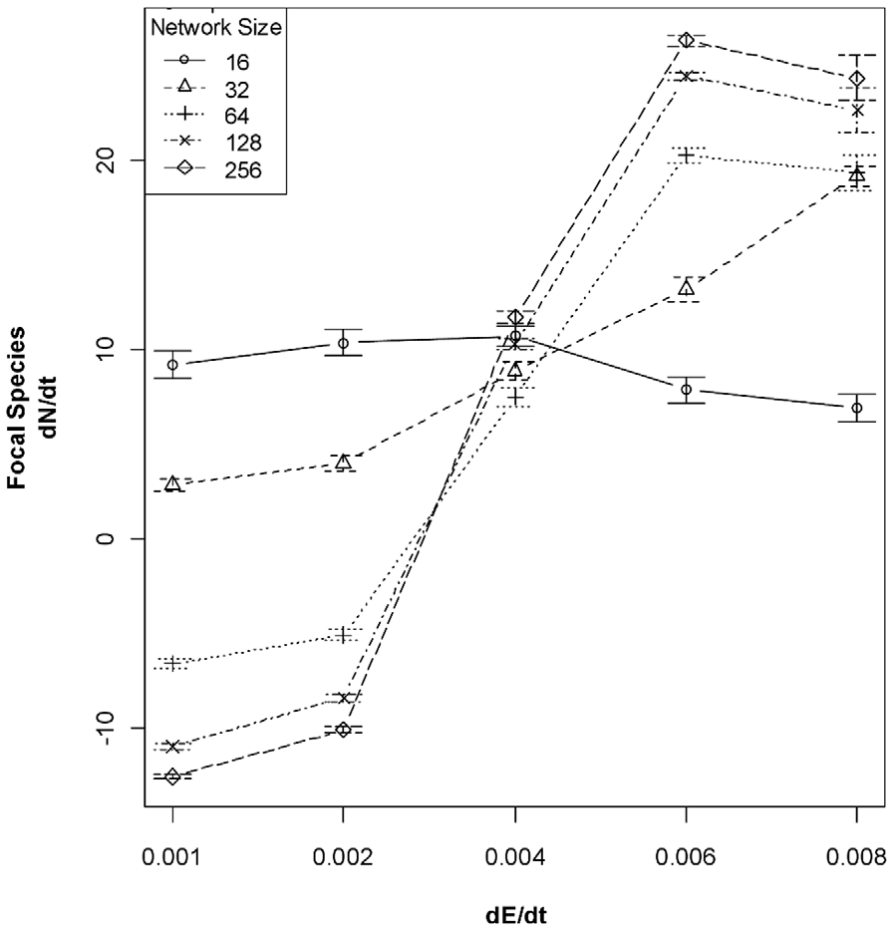
Effect of competitors (± 95% CI) with different genetic architectures and rates of environmental change (dE/dt) on the population growth rate of the focal species. The focal species possesses a fixed network size of 16 genes while competitors possess networks of size 16, 32, 64, 128, or 256 genes. Although the effect is not shown in this figure, the focal species has a fixed recombination rate (r = 0.05) and the competitor one of two rates (0.05 or 0.5). A strong interaction between dE/dt and network size is readily visible: larger competitor networks have a smaller and smaller impact on the focal species' dN/dt when the dE/dt is high. However, competitors with large networks have a progressively larger impact on the focal species' dN/dt when dE/dt is low. High dE/dt requires faster adaptation, and thus smaller networks have a competitive advantage, whereas the increased accuracy of larger networks is beneficial to the evolution of competitive ability at lower dE/dt. All networks are approximately equivalent at dE/dt = 4e^−3^.

A strong interaction between the rate of environmental change and size of the gene network underlying the ecologically-critical trait was apparent when two species compete. The first metric of this effect is the impact of the competitor on the focal species' population growth rate (dN/dt) in the first 20 generations of competition. The importance of the interaction between network size and dE/dt is readily apparent in Figure 1. The size of the competitor's gene network and the rate of recombination, conditional on interactions with the rate of environmental change (dE/dt), accounted for 79% of the variance in the focal species' dN/dt during the first 20 generations of competition (Table 1). This model possessed an Akaike's Information Criterion (AIC) score ≈120 points lower than the next-best model considered (see Methods). When the rate of environmental change is slow (<4e^−3^), a large-network competitor drives down the focal species' rate of population growth. However, when dE/dt is fast (>4e^−3^), the focal species' rate of population growth is positive and increases with the competitor's network size. Given the specifications of these simulations, all network sizes are approximately equivalent at dE/dt  = 4e^−3^.

**Table 1:** Gene network and environmental factors influencing the impact of a competitor on the focal species' population growth rate.

	*df*	% Variance Expl.	F-value	*p*-value
dE/dt	4	52	369.5606	<2.2e^−16^
Comp. Net. Size	4	0	2.6843	0.031
Comp. Recomb. Rate	1	1	36.6006	2.69e^−09^
dE/dt x Net.	16	23	40.7988	<2.2e^−16^
dE/dt x Recomb.	4	0	2.4316	0.047
Net. X Recomb.	4	2	14.7113	2.01e^−11^
dE/dt x Net. X Recomb.	16	1	1.4646	0.108

*dE/dt* is the rate of environmental change; *Comp. Net. Size* and *Net.* are the number of genes in the competitor's gene network; and *Comp. Recomb. Rate* and *Recomb.* are the competitor's recombination rate.

The basis of the different effects on the focal species' population growth rate can be inferred from the relative amounts of phenotypic and additive genetic variances (V_P_ and V_A_, respectively) of the two species conditional on dE/dt. The AIC-best model (ΔAIC ≈ 40) for explaining the focal species' dN/dt using variance components as predictor variables required knowing both the competitor's V_P_ and V_A_ and the interaction with dE/dt. The model explained 76% of variance in the focal species' dN/dt (Table 2). Although the competitor's V_A_ is not statistically significant on its own or at any given dE/dt, the interaction of V_A_ and dE/dt is significant over all levels. Both variance components tend to be lower for all networks larger than the focal species' network (Figure S1).

**Table 2:** Quantitative genetics variance components and environmental factors that influence the impact of a competitor on the focal species' population growth rate.

	*df*	% Variance Expl.	F-value	*p*-value
Comp. V_A_	1	0	0.7783	0.378
Comp. V_P_	1	2	41.9286	2.03e^−10^
dE/dt	4	55	340.7152	<2.2e^−16^
V_A_ x V_P_	1	0	2.4281	0.120
V_A_ x dE/dt	4	8	53.0199	<2.2e^−16^
V_P_ x dE/dt	4	11	71.5964	<2.2e^−16^
V_A_ x V_P_ x dE/dt	4	1	3.4333	0.009

*Comp. V_A_* (or *V_A_*) and *Comp. V_P_* (or *V_P_*) are the competitor's additive genetic and phenotypic variance, respectively; *dE/dt* is the rate of environmental change.

**Figure 2:**
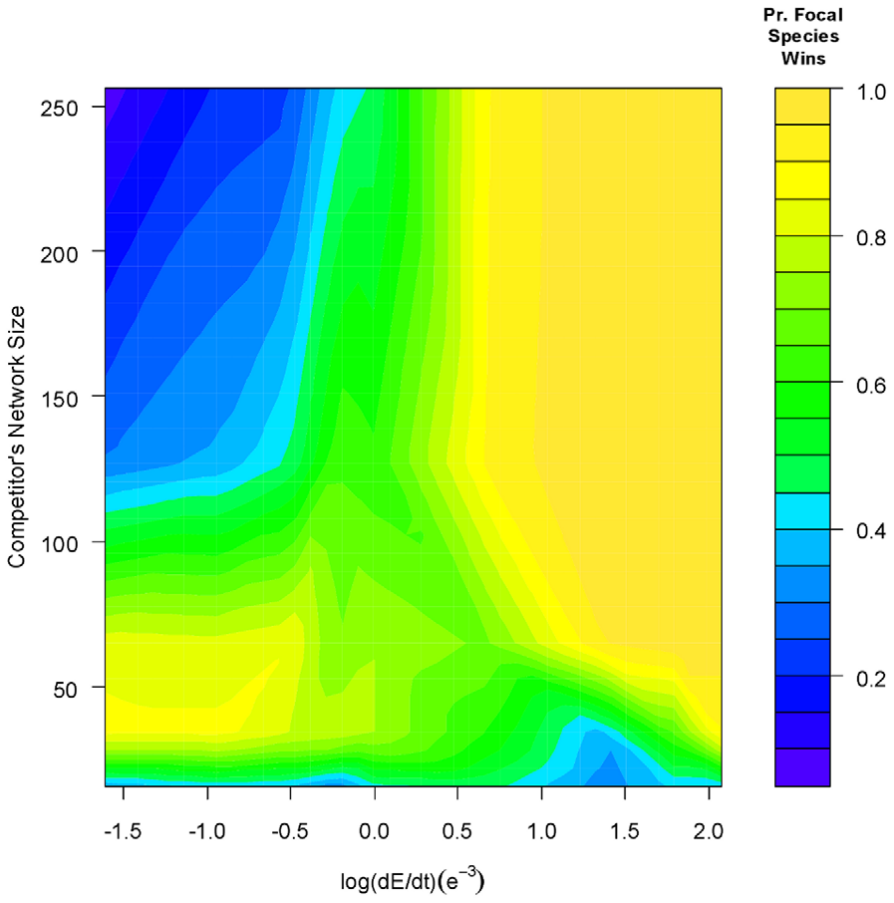
Probability that the focal species wins competition as a function of competitor network size and log(dE/dt). At slower rates of environmental change, the probability that the focal species will win declines with an increase in the size of the competitor's network. With the exception of a competitor with a 64-gene network, when the rate of environmental change is high, the probability of the focal species winning increases as the competitor's network size increases. 64-gene networks are never superior to the 16-gene network at the rates examined here. Note that this figure, produced using the *akima* package for R [54], interpolates data to produce the surface, whereas the predictor variables (network size, recombination rate, and dE/dt) are categorical in the simulations and statistical analyses.

The effects of differential adaptive ability on population growth rates during the initial competition phase are not completely transitive to predicting which species, the focal or competitor, ultimately wins. Because very few competitor wins were recorded at the rates of change examined in the first simulations (i.e., during the first 20 generations of competition), I extended the dE/dt landscape an order of magnitude slower (see Methods). The resultant descriptive pattern remains: smaller networks perform better than larger networks when dE/dt is high (and conversely when dE/dt is low), but dE/dt = 4e^−3^ is no longer the cutoff. Instead, smaller networks continue to perform well down to dE/dt = 1e^−3^, and only below that dE/dt do larger network competitors systematically win the competition (Figure 2). Although the focal species' population declines during the initial stages of competition, it appears that the larger-network competitor cannot sustain their higher level of adaptive accuracy and the focal species' population bounces back (Figures S2–S4). That is, although more accurate, the mean phenotype of the large-network species begins to lag too far behind the optimum (i.e., it is biased) and the lower-accuracy focal species gains an advantage. Two additional results stand out in Figure 2. First, the slightly lower than 50% probability of the focal species winning when the competitor's network is the same size as the focal species' derives from differences in recombination (see Methods). Second, a 64-gene network competitor never has an advantage over the 16-gene focal species network. Given the landscape of Figure 2, it appears that an even slower dE/dt could afford a 64-gene network an advantage over the focal species' 16-gene network, but I do not test that idea here. Over the landscape of dE/dt values examined, network size, the rate of environmental change, and the interaction of the terms explains a significant part of total model deviance in competitive outcomes (Table 3). The best model, on which Table 2 is based, possessed the lowest AIC by ≈ 40 points.

**Table 3:** Analysis of Deviance table for predicting the probability that the focal species wins competition.

	*df*	Deviance	Resid. *df*	Resid. Dev	*p*-value
NULL			3995	5148.4	
Comp. Net. Size	4	399.5	3991	4749.0	<2.2e^−16^
dE/dt	8	298.1	3983	4450.9	<2.2e^−16^
Comp. Recomb. Rate	1	0.1	3982	4450.8	0.737
Net. X dE/dt	32	766.1	3950	3684.6	<2.2e^−16^
Net. X Recomb.	4	16.7	3946	3668.0	0.002
dE/dt x Recomb.	8	23.3	3938	3644.7	0.003
Net. X dE/dt x Recomb.	32	87.1	3906	3557.6	5.5e^−7^

*Comp. Net. Size* and *Net.* are the number of genes in the competitor's gene network; *dE/dt* is the rate of environmental change; and *Comp. Recomb. Rate* and *Recomb.* are the competitor's recombination rate.

**Figure 3:**
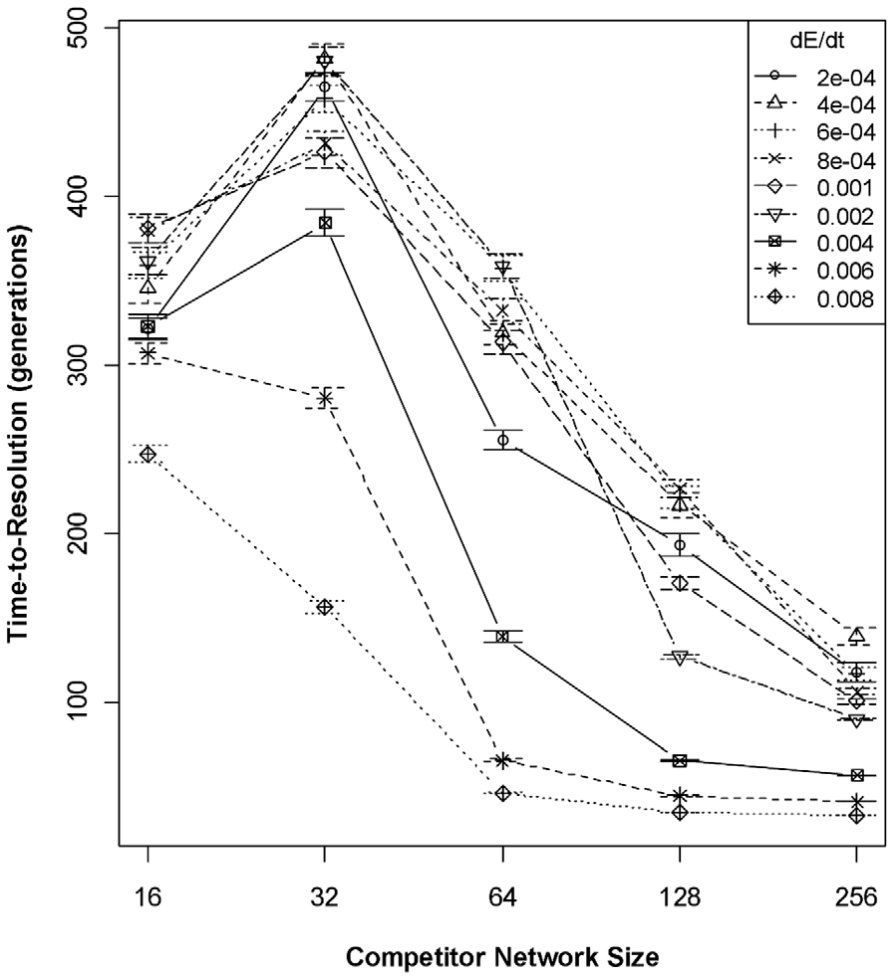
Effect (± 95% CI) of competitor's genetic architecture and the rate of environmental change (dE/dt) on the duration of competition. The time required for one of the two competing species to go to dominance (i.e., drive the other species extinct) in a single patch is largely a function of the relative difference in network sizes and the rate of environmental change (dE/dt). The focal species' genetic architecture is held constant (as in Figure 1) while the competitor species' genetic architecture varies. *Time-to-resolution* is the number of generations between the start of competition and the generation in which one species has gone extinct. Resolution occurs quickly when dE/dt is high—we quickly find that one species is not suited to the environment—whereas resolution takes considerably longer when dE/dt is low. Likewise, as the disparity between each species underlying network increases, the time-to-resolution declines. The lower persistence time for 16-gene network competitors is a result of the recombination rate treatment (see Methods).

The size of the competitor's gene network, the rate of environmental change, the competitor's recombination rate, and interactions were the major predictors of co-persistence times of the competing species (ΔAIC = 116.7), explaining ∼60% of the variance (Table 4). Larger differences between species' networks and higher rates of environmental change consistently decrease persistence times (Figure 3). In addition, differences in recombination rate tended to increase population persistence times, i.e., higher recombination affords an adaptive advantage at some network sizes. Note that this result speaks only to the fact that competition has ended, and not which species won; the adaptation speed/accuracy tradeoff is not apparent in time-to-resolution of competition.

**Table 4:** Factors influencing the time-to-resolution of competition when species differ in their genetic architecture.

	*df*	% Var. Explained	F-value	*p*-value
Comp. Net. Size	4	36	897.2191	<2.2e^−16^
dE/dt	8	19	238.5135	<2.2e^−16^
Comp. Recomb. Rate	1	0	1.6412	0.200
dE/dt x Comp. Net.	32	5	14.264	<2.2e^−16^
Net. x Recomb.	4	1	23.7332	<2.2e^−16^
dE/dt x Recomb.	8	0	1.6959	0.094
Net. X dE/dt x Recomb.	32	1	3.0367	2.89e^−06^

*Comp. Net. Size* and *Net.* are the number of genes in the competitor's gene network; *dE/dt* is the rate of environmental change; and *Comp. Recomb. Rate* and *Recomb.* are the competitor's recombination rate.

## Discussion

The interplay between genetic architecture, phenotypes, and evolutionary and ecological dynamics are complex, yet despite the rapid acceleration of biological research, a fundamental understanding of the interplay among these factors remains elusive. Progress is being made in refining the both the GPM and the PEM. Given this progress, we need sets of theoretical expectations to unite the constituent pieces. Here I have attempted a step in that direction with a set of simulations that span from the gene network underlying a quantitative trait to a simple two-species community in which interspecific competition occurs. Previous work that did not include competition suggested that specific characteristics of the genetic architecture of a trait could affect population dynamics when the environment suddenly shifts states or when it changes steadily through time [25], [31], [33]. One conclusion drawn from that work is that network size should be minimized, scale-free topology maintained, and intermediate network connectivity evolved in order to maximize adaptability. By including competition in the current model, I have increased the degree of realism and refined expectations of what we should observe when linking genotypes to ecological and evolutionary dynamics.

The major refinement of expectations is the trade-off between adaptive speed and adaptive accuracy, as revealed by the presence of a competitor and contrary to the expectation from single-species models. In rapidly changing environments the advantage of greater adaptive speed conferred by smaller networks is readily apparent. As the rate of environmental change slows, the probability of competitive superiority goes up with increasing network size. This is in contrast to single-species results, in which as rate of environmental change slows, populations of all network sizes converge on indefinite persistence time (see Figure 2 in [29]). In general, the lower V_A_ of larger networks is sufficient in slow-changing environments, while the lower V_P_ ensures that a large-network species is better adapted. In contrast, the higher V_A_ conferred by smaller networks is required in fast-changing environments; the small-network species does not adapt as well (higher V_P_), but it does not need to because the large-network species cannot adapt quickly enough. This is analogous to the importance of developmental accuracy as described by Hansen et al. [34].

The trade-off between adaptive speed and adaptive accuracy, in the context of the implications for the evolution of competition, has however not been previously recovered to my knowledge. Repsilber and colleagues [33] allowed their networks to evolve in size and discovered higher mean population fitness for single-species populations at different landscape heterogeneities, but did not consider >1 species in the landscape. The primary reason that the trade-off has not been previously recovered is that earlier work with competitors and an explicit GPM has focused on a single number of loci underlying a limiting trait. For example, Urban and de Meester used a model in which an ecologically-critical trait was underlain by 20 binary loci in each species [35]. If we consider an optimal phenotype of 0.53 (on the scale used by Urban and de Meester), the closest possible phenotype is 0.55 (11/20). Alternatively, if one species' GPM is defined by a 100-locus model, a phenotype of 0.53 is possible and would result in higher fitness. Given the joint processes of gene duplication and deletion [36]–[38], we can anticipate that certain traits may be underlain by fewer or additional genes, which should alter the speed and resolution of adaptation. These changes should then propagate up levels or organization to affect competitive dynamics as traits evolve, as shown here.

Convergence of genetic architecture—characteristics such as network size—becomes an equalizing mechanism [39] permitting long-term, essentially neutral, coexistence. In these simulations, as the difference in genetic architecture between two competing species increases, the persistence time of a two-species local community declines. Neither species can gain a distinct evolutionary-ecological advantage when genetic architectures are identical, and if an advantage is gained, it takes considerable time to evolve. An important caveat to the equalizing nature of genetic architecture change (by gene duplication and loss) is that differences in demographic parameters, such as generation time, could compensate for differences arising from gene regulatory network differences. For example, terHorst and colleagues showed that generation time differences between mosquito larvae and their protozoan prey altered eco-evolutionary dynamics [40]. However, if species are comparable in the variety of life history traits in addition to being limited by an analogous trait, then genetic architecture poses a tradeoff between speed and accuracy.

We may be able to link the network GPM concepts considered here to the models developed by Hairston and colleagues [7]. Their generalized model (their Eq. 3) incorporates rates of ecological and evolutionary change as the sum of two partial differential equations, the first describing the focal species' change relative to trait evolution and the second describing the focal species' change relative to non-evolutionary demographic factors. We should expect that large network differences between competing species increases the relative role of evolution in total ecological change. This is conditional on the relative differences in demographic parameters of the competing species, however: if those differences are greater than even a large network difference, then demographic differences would still play a larger role than evolutionary differences. With this condition in mind, we can hypothesize that we should find larger differences in the networks underlying competition-critical traits in systems where evolutionary change is dominant, but more similar gene networks where demographic changes drive the system.

The results of these simulations suggest a further hypothesis: that communities composed of species with similar genetic architectures (for limiting traits) give rise to neutral community dynamics, whereas differences in genetic architecture give rise to species sorting dynamics. The identical evolutionary potential of species is, in fact, an assumption of Hubbell's neutral theory [41]. Conversely, we can hypothesize that the prevalence of niche-driven species sorting in many ecological communities [32] could be a result of differences in adaptive potential resulting from differences in the genetic architecture of ecologically-critical traits. That is, when considering the genetic architecture of ecologically-critical traits as evolving networks, a novel axis of species sorting [42], [43] seems to emerge. Classical species sorting considers traits as fixed, but these simulations show that traits can evolve and species assort in a single patch according to the network best-suited to particular rates of environmental change and the competitive challenge posed by another species. The degree to which this axis of species sorting occurs will depend on the relative rates of dispersal among a set of patches, and the heterogeneity of the patches, in a metacommunity.

How do these results compare to the real world? The short answer is, we don't know. This is driven in large-part by the fact that the tools necessary for elucidating the GPM are recent developments, and, at this time, still relatively expensive. I have proposed that a given trait in different species may be underlain by different size networks and that these differences can drive evolutionary ecological patterns such as competitive dynamics. An alternate hypothesis—and perfectly reasonable in the absence of empirical data—is that any particular challenge requires approximately the same size network regardless of the species in question and its evolutionary history. For example, perhaps osmoregulation requires, say, 250 genes (or, more correctly, the products of 250 genes and their associated regulatory loci), and any differences in adaptive capacity are due solely to specific sequences and gene regulation. We might even expect such a pattern to emerge: as discussed above, given sufficient time for gene duplication and loss [36], [37], trait genetic architecture should converge as an equalizing mechanism [39]. Ultimately, either result—very similar network sizes or different network sizes—from empirical data would be interesting and informative, even if the latter makes the results herein irrelevant.

In addition to our lack of data to confirm this work, we have to consider that these simulations, like all models, are simplifications of reality. The basic caveats to the research here largely follow the caveats of Malcom [25]: Boolean regulatory networks gloss over real differences of gene functions, the details of which are interesting and may have important ramifications. The networks I use here are simplified in that each gene is regulated by a single upstream factor, whereas real genes are often multiply regulated. We have ample evidence of widespread pleiotropy between networks [44]–[46], and the traits that these linked networks underlie may be under different selection regimes, which alters the efficiency of natural selection. Lastly, the competition scenario considered here is greatly simplified, and other (non-network) research has shown the multi-species and multi-trophic scenarios can alter eco-evolutionary trajectories in unpredictable ways [47]. There are numerous directions that future research could take. First and foremost, empirical support (or rejection) of the basic assumptions in this purely theoretical paper needs to be gathered; for example, do different species possess different size networks for the same trait? Second, because we know both phenomena are widespread, incorporating pleiotropy and plasticity in similar, network-based models would increase realism and may further refine our theoretical expectations. Including >2 species, and/or two or more trophic levels, with the GPM defined as complex networks could further refine our expectations of the links across the GEM.

There are two main conclusions from this research. First, there is an adaptation speed-accuracy tradeoff conferred by network size (and to a lesser extent, recombination). This tradeoff allows species with slow-evolving traits (i.e., large underlying networks) to out-compete species with fast-evolving traits (i.e., smaller networks) by virtue of increased adaptive accuracy. Second, the trade-off is contingent on the rate of change of the environmental variable to which the trait maps. Together, these results suggest that ecological interactions such as competition should contribute to the shaping of gene networks underlying quantitative traits. Therefore, not only should knowledge of the ecological interactions of a study species contribute substantially to our expectations of what should be observed when the GPM is investigated, but knowledge of the GPM may provide important information about why certain ecological patterns or processes are observed.

## Materials and Methods

### Gene Network Model

I focus on individuals of two species competing in a single patch with an environmental variable that fluctuates through time at a variety of rates. Individuals of either species possess a single quantitative trait that maps to the quality of the limiting resource (discussed in detail below). The trait is encoded by a directed Boolean network of 16, 32, 64, 128, or 256 genes, the state of each determined dynamically (see below). The topology of the network is initiated as either random (no preferential attachment) or scale-free (with preferential attachment) in its out-degree distribution [48]. Randomly-connected networks show an approximately Poisson degree distribution, whereas scale-free networks exhibit an power law degree distribution [49]. I use a lottery model algorithm to form the scale-free networks, i.e., the probability of an existing gene acquiring a connection to a new gene is proportional to the number of existing connections [49].

**Figure 4:**
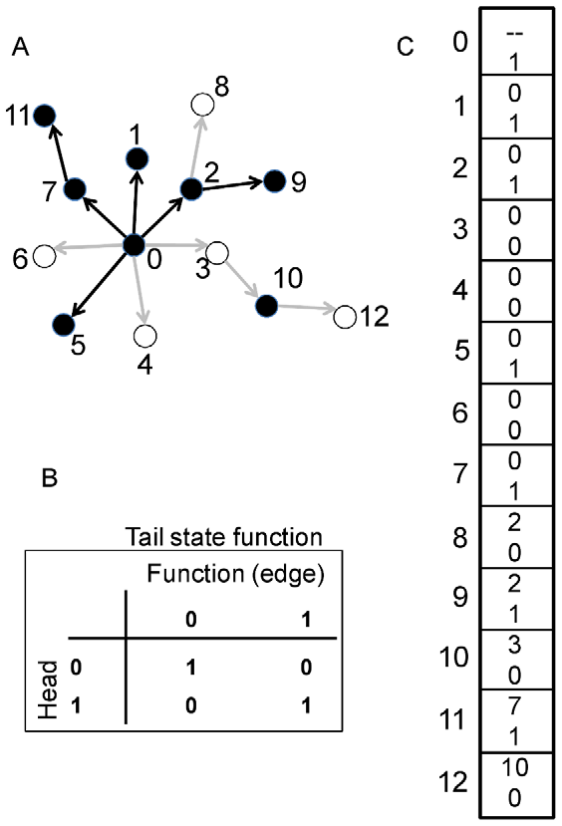
An example network, functional map, and chromosome. Panel A shows an example 13-gene Boolean network. Black nodes are up-regulated (“on”; state = 1) genes and white nodes are down-regulated (“off”; state = 0). If an edge connecting two nodes is black, the “head” gene (upstream) activates the “tail” gene (downstream), and if an edge is gray, the head represses the tail gene. Panel B provides the functional map; for example, if the head gene is “off” and the edge connecting the head and tail genes is an activator, then the tail gene is off (upper-right quadrant). Panel C shows the chromosome corresponding to the network in Panel A. Each block represents a gene (numbers along the left-hand side); within each block, the top number defines the “head” (i.e., immediately-upstream) gene while the bottom number defines the functional relationship (e.g., if 0, then the head gene is a repressor).

At the start of a run, every individual's network is randomly determined (as guided by the constraints of topological specification). With these relatively small populations, it is very unlikely that any two individuals possess the same exact network at simulation initiation. The binary state [0, 1] of each gene in the network except the upstream-most is determined by comparing the state of the gene immediately upstream to the functional relationship of the gene pair (Figure 4a, encoded by chromosome of 4c). The state of the upstream-most gene is determined randomly for each individual at simulation initiation, and is then inherited for subsequent generations. Some genes may act as repressors and others as activators, and the state of the downstream gene is determined by the match or mismatch between the state of the upstream gene and the function (Figure 4b). For example, if the upstream gene is “on” (state  = 1) and is a repressor (function  = 0), then the downstream gene takes the “off” state (state  = 0). Alternatively, if the upstream gene state is 0 and it is a repressor, then the downstream gene takes the “on” state. Each gene except the basal-most has a single input to ease computational requirements (the number of calculations increases according to 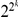 with *k* inputs [29]), but may have one or more outputs (i.e., may be pleiotropic). All network information is stored on a single chromosome consisting of two parts (Figure 4c). First, the topology is defined by a “tails list” of the downstream genes; the “heads list” (the controlling, upstream genes) is inferred from the index position of each tail list element. The relationship between heads and tails genes is randomly determined at the start of a simulation run, but, as noted above, the out-degree distribution is constrained by the scale-free versus random topological assignment. Figure 4a is an example 13-gene network whose states have been calculated given the information from the chromosome in Figure 4c.

Each individual's phenotype is determined by summing the states of all terminal genes in the network, i.e., genes with out-degree = 0, and scaling the value to the range of the environment ( = 140). So, for example, the network in Figure 4a possesses eight terminal genes, four of which are “on”, thus the individual possesses a phenotype of 70 ( =  (140/8) * 4). I am thereby assuming that there are no biochemical limits given a particular network size; individuals with a 16-gene network can approximate a phenotype of 140, as can individuals with a 256-gene network. The consequence for this re-scaling is that smaller networks have lower resolution than larger networks, which is a reasonable assumption given that dividing any particular task among fewer actors will result in lower overall accuracy. I stored the phenotypes of each individual's parents and used mid-parent regression to estimate the trait's heritability in the population. Additive genetic variance was derived by multiplying the phenotypic variance by the heritability.

Each individual's phenotype is translated to a fitness relative to the environment using a Gaussian function of the form,



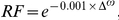
where Δ is the absolute value of the difference between the environment and the individual's phenotype, and ω is a value that changes the breadth of the selection function. I varied ω from 1.5 (high tolerance for a phenotype-environment mismatch) to 2.5 (low tolerance for a phenotype-environment mismatch) in the simulations. In this way I assume that the environmental effect is absolute and the phenotypic variance of the population plays no role in how an individual is selected. Each individual's *RF* does not affect the number of offspring produced, but does affect the probability that an individual will survive to reproduce.

Individuals are sexually-reproducing hermaphrodites who mate at random. The number of offspring from a mating is determined by drawing a random value from a Poisson distribution with λ = 1.5. Gametes undergo recombination during a diploid meiotic stage to create an offspring chromosome that is a mixture of parental alleles, which in this model are the tails list and the functional relationships. The first element of the offspring chromosome is chosen from the first element of one parent, then subsequent elements are taken from the same parent until a random uniform number less than the recombination rate (*r* = 0.05 or 0.5) is drawn, at which point the element is drawn from the opposite parent. This continues the length of the chromosome. Mutation, as determined by testing a uniform random number against the mutation rate (1e^−3^ or 1e^−5^) for each chromosomal element, occurs after the new chromosome is created. Although these mutation rates appear high, as noted by Frank [30], because the trait is directly related to fitness, the effective mutation rate is about one order of magnitude lower. All mutations are non-synonymous and may affect either the controlling function of a gene (an activator mutates to suppressor) or the relationship to another gene (i.e., alter network topology).

Death occurs after reproduction in three stages. First, all parents are killed to prevent over-lapping generations. Next, the new generation is culled according to each individual's relative fitness: if the *RF* is less than a uniform random number, then the individual dies. Last, a carrying-capacity is enforced by randomly killing individuals to bring the population below K = 500.

### Competition Simulations

As discussed in the Introduction, the two competing species are co-limited in this model. First, the resource occurs at a given quantity that limits the total number of individuals in a patch, and the two species are effectively neutral with respect to capitalizing on quantity (i.e., their requirement and impact vectors are identical [32]). Second, the resource has a quantitative value for quality, such as palatability, to which the competing species must adapt in order to maximize their fitness. The quantitative trait, whose value is determined by the gene network, maps to this resource quality. Specifying competition in this way stabilizes the population dynamics relative to a system in which the primary resource is depleted. Note, however, that this does not permit exploring the effects of over-exploitation, which could alter competitive dynamics.

An initial canalization period is important for reducing excess initial phenotypic and genotypic variance. Simulations are initiated with each species in its own patch, and competition occurs in a third patch. The environmental variable is initialized at the same value ( =  70) and changes at the same rate (8e^−3^ to 2e^−4^ units per generation; details below) in all three patches. A single dispersal event occurs after the 20-generation canalization period and 200 randomly-chosen individuals of each species—which are as well-adapted to the same environment, insofar as their genetic architecture allows—are moved to the third patch. Any individuals not selected to disperse are killed.

I ran two sets of simulations. In the first, I examined the effect of the competitor on the focal species' dN/dt over the first 20 generations of competition, i.e., up through generation 40. These simulations were full-factorial for genetic architecture of the competitor (five network sizes, two network topologies, two recombination rates, and two mutation rates) and six rates of environmental change (dE/dt = 8e^−3^, 6e^−3^, 4e^−3^, 2e^−3^, or 1e^−3^), replicated 40 times for each combination.

After the first set of simulations had been completed and analyzed, and no effects of network topology or mutation rate were observed, I ran a new set of simulations. These were full-factorial for five network sizes, two recombination rates, and five rates of environmental change, as above. Analysis of this initial set of full runs showed that even though the dN/dt values were depressed at low dE/dt, the focal species still typically won competition. I then ran another set of simulations with slower dE/dt ( = 8e^−4^, 6e^−4^, 4e^−4^, or 2e^−4^) and all competitor genetic architecture treatments. Both of these sets of runs were represented by 40 replicates of each treatment combination.

### Analysis

For all analyses, except when noted otherwise, the predictor variables are factors rather than continuous values. Thus, even though some figures suggest non-linear models may be appropriate, they are not necessary given the structure of the simulations and analysis. A summary of the models considered, and for which AIC was calculated, is provided in Table 5. Standard AIC, as opposed to AIC_C_, was used because of the large sample sizes for the simulations. All simulations were run in NetLogo 4.1 [50]. I used R 2.10 [51] for statistical analysis, and Akaike's Information Criterion (AIC) for model selection [52].

**Table 5:** Models considered for the initial competition, winner of competition, and time-to-resolution of competition analyses.

Analysis	Model	Model Description
Initial Competition	IN1	dN/dt = dE/dt x n x sf x μ x r
(network characteristics)	IN2	dN/dt = dE/dt x n x r
	IN3	dN/dt = dE/dt x n
	IN4	dN/dt = dE/dt + n + r
Initial Competition	IV1	dN/dt = compV_A_ x compV_P_ x dE/dt
(variance components)	IV2	dN/dt = compV_A_ x dE/dt
	IV3	dN/dt = compV_P_ x dE/dt
	IV4	dN/dt = focV_A_ x focV_P_ x compV_A_ x compV_P_ x dE/dt
Competition Winner	CW1	Pr(focal_sp_wins) = comp_n x comp_r x dE/dt
	CW2	Pr(focal_sp_wins) = comp_n x dE/dt
	CW3	Pr(focal_sp_wins) = dE/dt
	CW4	Pr(focal_sp_wins) = comp_n + comp_r + dE/dt
Time-to-Resolution	TR1	log(time-to-resolution) = comp_n x comp_r x dE/dt
	TR2	log(time-to-resolution) = comp_n x dE/dt
	TR3	log(time-to-resolution) = comp_n
	TR4	log(time-to-resolution) = comp_n + comp_r + dE/dt

*dN/dt* is the focal species' rate of population change; *dE/dt* is the rate of environmental change; *n* is the size of the competitor's gene network; *sf* is the topology (e.g., *s*cale-*f*ree) of the competitor's network; μ is the competitor's mutation rate; *r* is the competitor's recombination rate; and these abbreviations are combined for brevity in some model descriptions.

To analyze the first set of simulations, I estimated the focal species' dN/dt during the 20 generations following the start of competition of each run using a basic linear model of population on time. The slope of each regression was stored and used as the response variable in the models described under *Initial Competition* in Table 5. I used two sets of predictor variables to examine the determinants of focal species' dN/dt, the first focused on network characteristics and the second focused on quantitative genetics variance components (V_P_ and V_A_). This latter analysis was designed to link the simulations to the classical understanding of evolutionary dynamics, but it is important that the variance components are emergent properties of the networks and populations, rather than being specified a priori.

I considered two response variables for the second set of simulations. First, I extracted the winner of each simulation run; if the run lasted 1,000 generations, then the species with the larger population at the last time step was called as the winner. Second, I extracted the time (i.e., generation) of the end of each simulation run; a slight skew to the time-to-resolution data required a log transformation to ensure normally-distributed residuals. I used a generalized linear model with a binomial distribution and logit link function [53] to relate the network and dE/dt predictor variables to the probability that the focal species won the competitive bout (Table 5, *Competition Winner*). Figure 2 was generated using the *akima* package for R [54] and treats the predictor variables as continuous values for interpolation purposes. However, predictors were factors in the analysis presented in Table 3. I used an OLS linear regression to relate network characteristic and dE/dt predictor variables to log-transformed time-to-resolution (Table 5, *Time-to-Resolution*).

## Supporting Information

Figure S1 Comparison of focal species' and competitors variance components at the start of competition. There is no discernible pattern to VA and VP in the focal species (left panels), but the competitor's VA and VP decline with increasing size of the competitor's network (right panels). Larger-network competitors cannot persist in fast-changing environments, suggesting that VA ≥20 is required to keep up with the changing environment at the higher dE/dt. The lower VP affords a competitive advantage (i.e., more individuals are closer to the optimal trait value) when networks are large and dE/dt is slow.
(0.45 MB TIF)

Figure S2 Mean VA of the focal species (solid line) and competitor (dashed line) over the course of competition. These five panels are from runs at dE/dt = 4e-3, 2e-3, and 1e-3, where the initial impact of the competitor is to suppress the focal species, but eventually the focal species tends to recover and win competition. Note these plots are averaged over all three rates of environmental change (dE/dt). The solid, vertical bars in each plot indicate the average end-of-competition time for each network size treatment. The end of competition occurs most-quickly when the difference in VA between species is most evident, and persistence is highest throughout when VA is similar. Importantly, although VA quickly becomes similar (ca. 100 generations), the 16-gene competitor typically wins (see Figure 2). See Figure S4 for a partial further explanation.
(0.55 MB TIF)

Figure S3Mean VP of the focal species (solid line) and competitor (dashed line) over the course of competition. These five panels are from runs at dE/dt = 4e-3, 2e-3, and 1e-3, where the initial impact of the competitor is to suppress the focal species, but eventually the focal species tends to recover and win competition. The solid, vertical bars in each plot indicate the average end-of-competition time for each network size treatment. Note these plots are averaged over all rates of environmental change (dE/dt). Longer persistence time is associated with minimized difference in 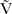
P, but even when VP is similar, the competitor loses (see Figure 2). See Figure S4 for a partial further explanation.(0.33 MB TIF)

Figure S4 Mean difference of the average phenotype minus the environmental value of the focal species (solid line) and competitor (dashed line) over the course of competition. At the dE/dt considered here, the focal species should lose competition-at least against a larger-network competitor-because the focal species' dN/dt is much lower than when competing against a 16-gene species (see Figure 1). In these plots, however, we see that the difference between the optimal trait value (i.e., the environmental value) and the population mean tends to be much larger for the competitor (at least for 64- to 256-gene competitors). That is, although the competitor is more accurate, it is more biased, and therefore eventually loses the competition.
(0.31 MB TIF)
