# Supplementary figures and images for: Evolution of Competitive Ability: An Adaptation Speed vs. Accuracy Tradeoff Rooted in Gene Network Size
Source: PLoS One. 2011 Apr 25;6(4):e14799. doi: 10.1371/journal.pone.0014799 (PMC3081808; doi:10.1371/journal.pone.0014799)

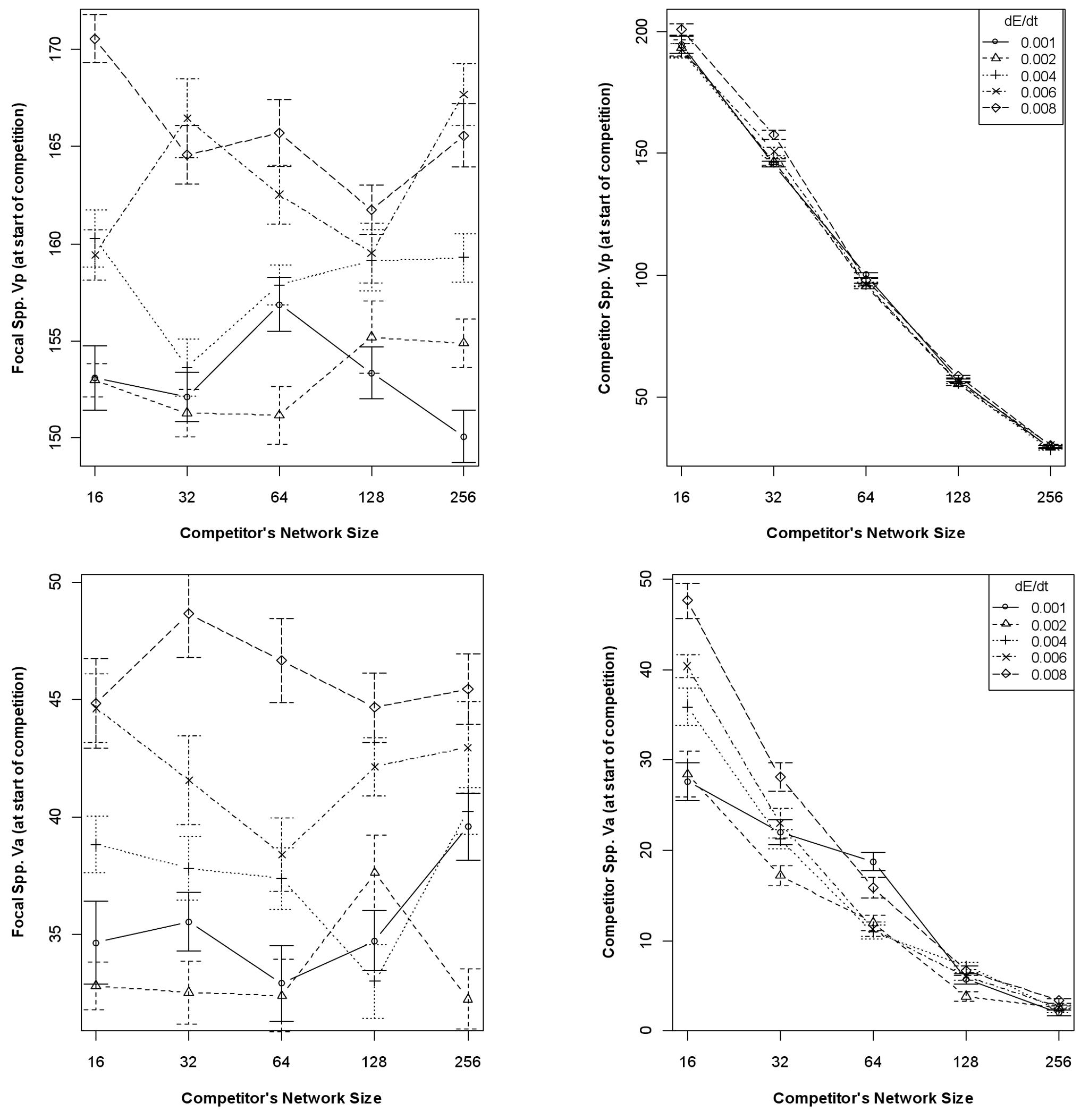

Supplement: Figure S1 — Comparison of focal species' and competitors variance components at the start of competition. There is no discernible pattern to VA and VP in the focal species (left panels), but the competitor's VA and VP decline with increasing size of the competitor's network (right panels). Larger-network competitors cannot persist in fast-changing environments, suggesting that VA ≥20 is required to keep up with the changing environment at the higher dE/dt. The lower VP affords a competitive advantage (i.e., more individuals are closer to the optimal trait value) when networks are large and dE/dt is slow. (0.45 MB TIF) [file pone.0014799.s001.tif]

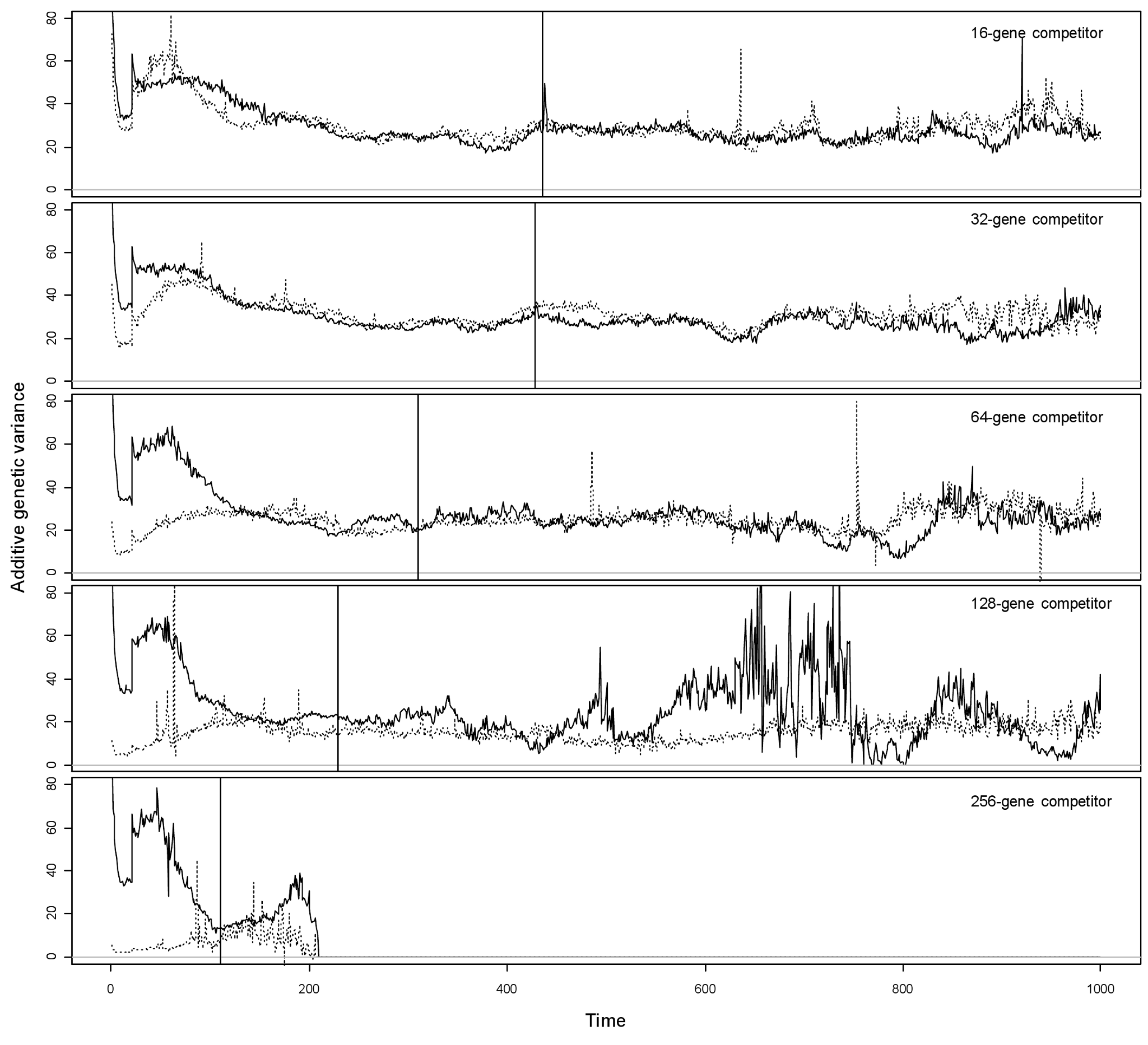

Supplement: Figure S2 — Mean VA of the focal species (solid line) and competitor (dashed line) over the course of competition. These five panels are from runs at dE/dt = 4e-3, 2e-3, and 1e-3, where the initial impact of the competitor is to suppress the focal species, but eventually the focal species tends to recover and win competition. Note these plots are averaged over all three rates of environmental change (dE/dt). The solid, vertical bars in each plot indicate the average end-of-competition time for each network size treatment. The end of competition occurs most-quickly when the difference in VA between species is most evident, and persistence is highest throughout when VA is similar. Importantly, although VA quickly becomes similar (ca. 100 generations), the 16-gene competitor typically wins (see Figure 2). See Figure S4 for a partial further explanation. (0.55 MB TIF) [file pone.0014799.s002.tif]

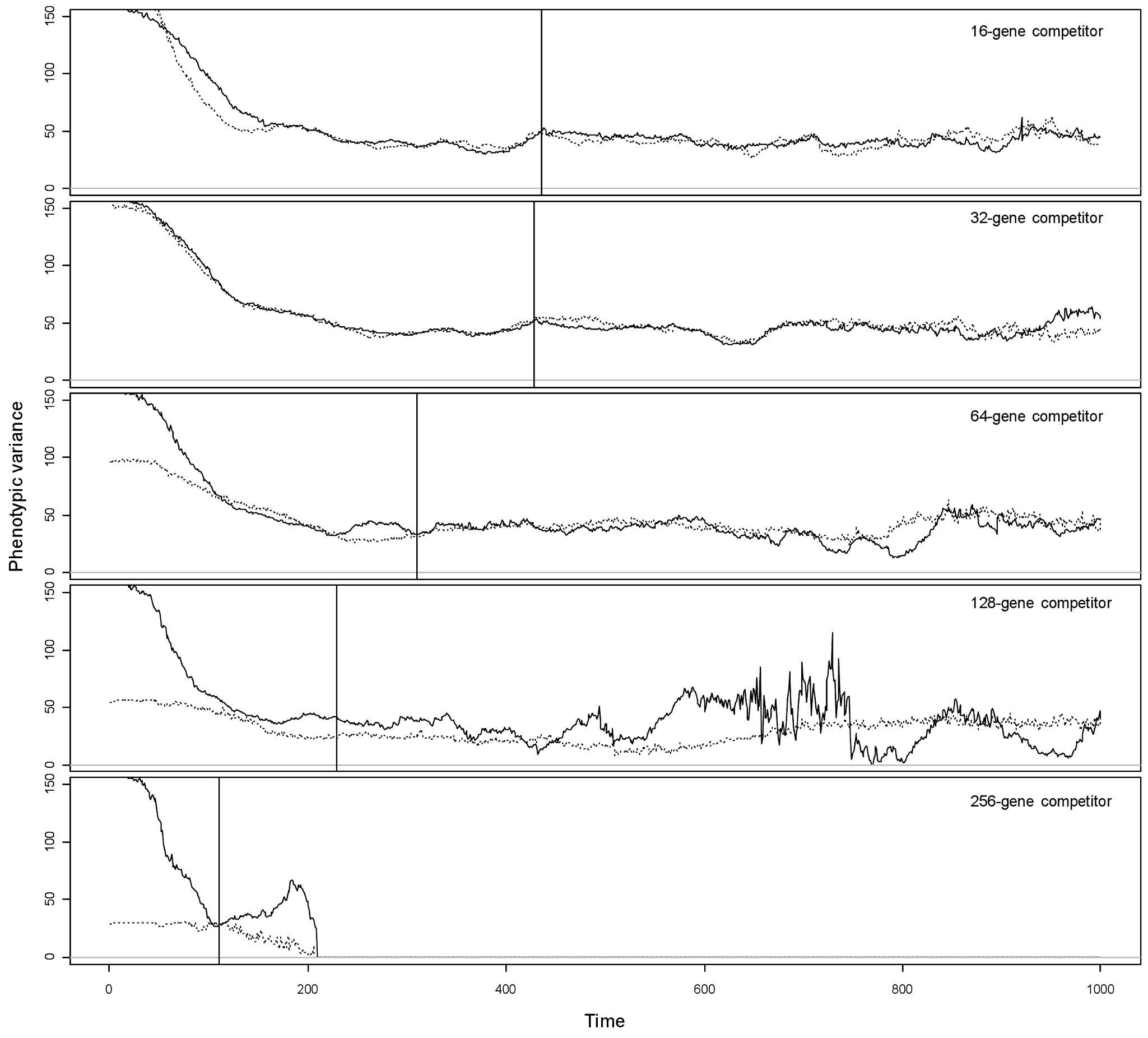

Supplement: Figure S3 — Mean VP of the focal species (solid line) and competitor (dashed line) over the course of competition. These five panels are from runs at dE/dt = 4e-3, 2e-3, and 1e-3, where the initial impact of the competitor is to suppress the focal species, but eventually the focal species tends to recover and win competition. The solid, vertical bars in each plot indicate the average end-of-competition time for each network size treatment. Note these plots are averaged over all rates of environmental change (dE/dt). Longer persistence time is associated with minimized difference in P, but even when VP is similar, the competitor loses (see Figure 2). See Figure S4 for a partial further explanation. (0.33 MB TIF) [file pone.0014799.s003.tif]

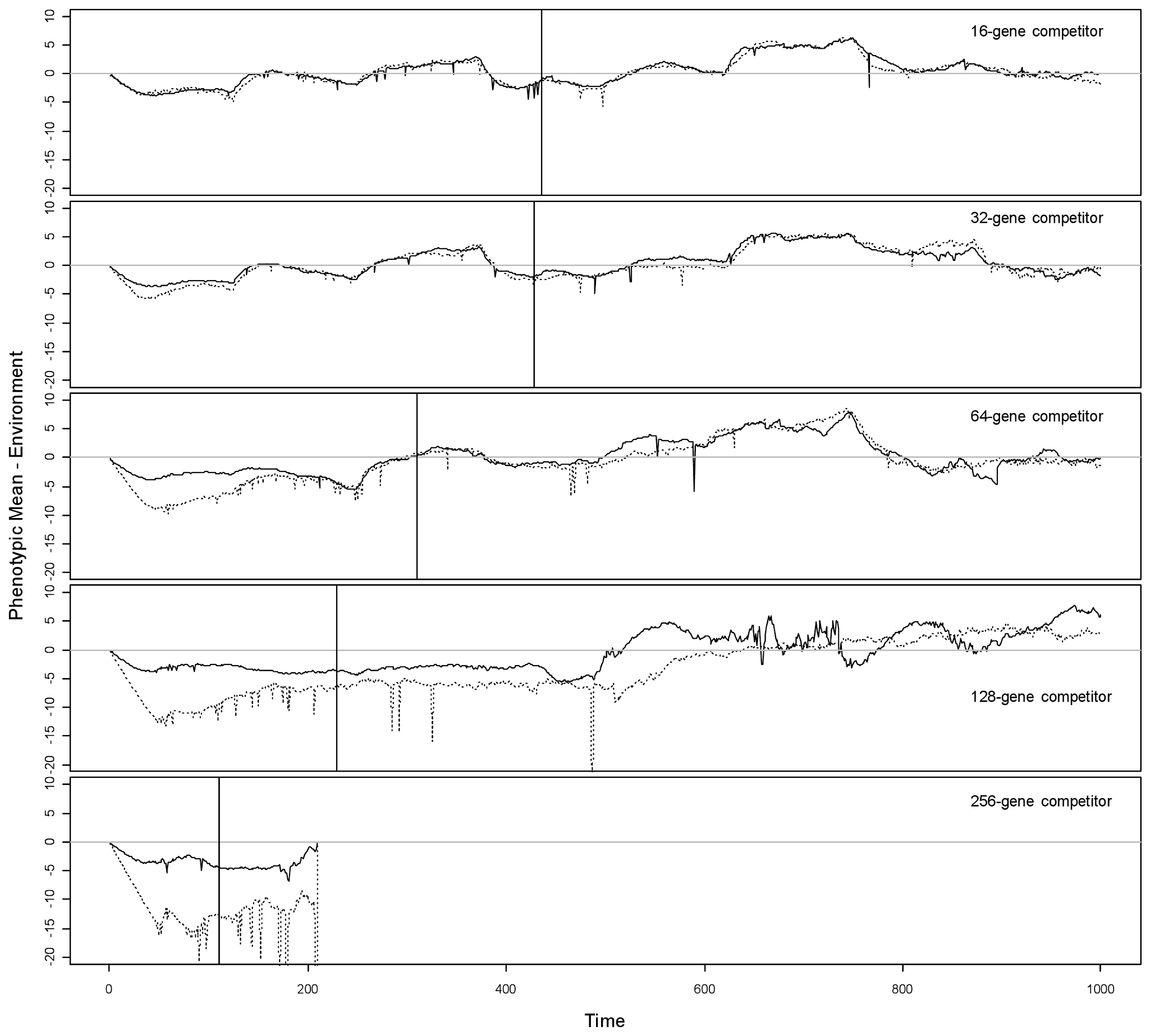

Supplement: Figure S4 — Mean difference of the average phenotype minus the environmental value of the focal species (solid line) and competitor (dashed line) over the course of competition. At the dE/dt considered here, the focal species should lose competition-at least against a larger-network competitor-because the focal species' dN/dt is much lower than when competing against a 16-gene species (see Figure 1). In these plots, however, we see that the difference between the optimal trait value (i.e., the environmental value) and the population mean tends to be much larger for the competitor (at least for 64- to 256-gene competitors). That is, although the competitor is more accurate, it is more biased, and therefore eventually loses the competition. (0.31 MB TIF) [file pone.0014799.s004.tif]
